# Psychiatric nurses’ understandings and attitudes toward the participation of patients with mental disorders in shared decision-making: a phenomenological study

**DOI:** 10.3389/fpsyt.2026.1864939

**Published:** 2026-07-17

**Authors:** Yan Han, Tiantian Mou, Yan Qiao, Qian Wang

**Affiliations:** 1 Department of Nursing, Huai’an No. 3 People’s Hospital, Huai’an, Jiangsu, China; 2 Department of Geriatric Psychiatry, Huai’an No. 3 People’s Hospital, Huai’an, Jiangsu, China

**Keywords:** closed psychiatric wards, mental disorders, patient participation, phenomenological research, psychiatric nurses, shared decision-making

## Abstract

**Background:**

Shared decision-making (SDM) is increasingly promoted in mental health care, but its meaning and feasibility remain contested in closed psychiatric wards where autonomy, fluctuating decision-making capacity, safety, coercion, family involvement, and clinical responsibility intersect. This study explored how psychiatric nurses understand patient participation in SDM and how they position themselves toward its implementation in this setting.

**Methods:**

A phenomenological qualitative design was adopted and reported with reference to COREQ. Sixteen psychiatric nurses from closed acute, chronic, and rehabilitation wards of a tertiary psychiatric hospital in Jiangsu Province, China, were purposively sampled between April and June 2024. Seventeen semi-structured face-to-face interviews were conducted because one participant completed a follow-up interview for clarification. Interviews were audio-recorded, transcribed verbatim, and analyzed using Colaizzi’s seven-step method with team-based coding, reflexive memoing, and an audit trail.

**Results:**

Five overarching themes emerged. In the domain of understandings, nurses endorsed the value of SDM but interpreted it through situated clinical reasoning, including conflating SDM with informed consent, viewing patients’ decision-making capacity as constrained by illness phase and risk, anticipating workload and risk pressure, and positioning nurses mainly as intermediaries rather than co-decision-makers. In the attitudinal domain, support, cautious neutrality, and opposition were better understood as conditional positions along a continuum shaped by patient acuity, ward culture, institutional safeguards, family authority, and perceived medico-legal risk.

**Conclusion:**

Psychiatric nurses did not simply accept or reject SDM; rather, they interpreted it through the practical tensions of closed psychiatric care. Promoting SDM in this context requires capacity-sensitive communication, structured risk and capacity assessment, explicit ward-level procedures, interprofessional case discussion, family negotiation strategies, and institutional safeguards that integrate patient participation with safety and legal responsibility.

## 1 Introduction

Patients with mental disorders often experience fluctuating cognition, affect, behavior, insight, and social functioning, which makes participation in health-care decision-making especially complex. In psychiatric settings, decisions about medication, nursing interventions, hospitalization, restriction, crisis management, and rehabilitation are frequently made under conditions of uncertainty and risk. Shared decision-making (SDM) has therefore become a key concept for reconciling clinical expertise with service users’ lived experience, preferences, and values, but it cannot be understood as a simple transfer of decision authority from professionals to patients (1–4).

Conceptually, SDM refers to a collaborative process in which clinicians and patients share information, discuss reasonable options, deliberate about benefits and burdens, and arrive at a decision that is clinically appropriate and responsive to the patient’s values (1, 2). Patient participation denotes the broader involvement of patients in expressing needs, asking questions, clarifying preferences, and contributing to care planning, whereas informed consent refers more narrowly to disclosure, comprehension, voluntariness, and agreement before a specific intervention. Autonomy is likewise not identical to isolated independent choice; in psychiatric care, it is often relational and may require communication support, staged deliberation, and involvement of trusted others. Existing evidence suggests that SDM in psychiatry can strengthen therapeutic relationships, engagement, satisfaction, and the alignment between care plans and patient priorities (5–8). Qualitative studies also show that participation in decision-making may be experienced by service users and families as a marker of dignity, trust, and recovery-oriented care (8–12).

At the same time, SDM in psychiatry is shaped by variables that are less prominent in many other specialties, including clinical risk level, symptom acuity, fluctuating decision-making capacity, involuntary treatment, ward restriction, family involvement, and the urgency of intervention. Decision-making capacity is understood in this study as task-specific, situation-dependent, and potentially fluctuating rather than as a stable global attribute attached to a diagnosis (13). Mental health professionals may therefore support SDM as an ideal while remaining uncertain about when, how, and to what extent patients with psychosis, severe mood symptoms, suicidal risk, aggression risk, or impaired insight can participate in particular decisions (14–17). Other barriers include insufficient training, limited decision aids, rigid routines, fear of accountability, and a persistent paternalistic culture in inpatient psychiatry (14–17). The implementation question is therefore not whether SDM is universally desirable in the abstract, but how participation can be adapted to specific decisions while maintaining clinical responsibility and safety.

Nurses occupy a particularly important position in this context. Compared with psychiatrists, psychiatric nurses usually spend more continuous time with hospitalized patients, observe symptom change across the day, negotiate treatment adherence at the bedside, manage distress and conflict, and often become the first professionals to hear patients’ doubts, wishes, or refusals. For this reason, nurses are not peripheral to SDM; they are central to whether it is enacted in ward life. Recent literature has highlighted that nursing staff can facilitate SDM by translating medical information, eliciting patient concerns, coaching patients to express preferences, and maintaining therapeutic dialogue across repeated interactions (18–21). Conversely, if nurses interpret their role narrowly as information transmission or order execution, the practical space for SDM may shrink substantially even when institutional discourse appears supportive.

Within psychiatric hospitals in China, interest in SDM has grown, but evidence regarding nurses’ understanding of patient participation remains limited, especially in closed wards. The Chinese psychiatric care context is relevant because professional responsibility, family participation, guardianship practices, involuntary admission standards, and risk management are embedded in specific legal and cultural arrangements. The Mental Health Law of the People’s Republic of China emphasizes protection of the lawful rights and interests of persons with mental disorders while also providing legal conditions for involuntary hospitalization when risk thresholds are met (22). In practice, families often play a substantial role in treatment communication and decision support, which may facilitate care continuity but may also complicate the boundary between patient autonomy and family authority (4, 19, 22). Existing studies indicate that Chinese mental health professionals recognize the promise of SDM but remain uncertain about its operational meaning, applicability in severe mental illness, and compatibility with ward routines and family-influenced treatment cultures (4, 19). Broader implementation research likewise shows that practitioners endorse SDM abstractly while struggling with feasibility, tools, and workflow integration in everyday practice (21, 23, 24). Studies of patient preference and inpatient drivers further indicate that many service users want more influence than they currently receive, even in psychiatric settings (9, 25), and conceptual work in mental health ethics warns against equating vulnerability with global incapacity (26).

The present study addressed this gap through phenomenological interviews with psychiatric nurses from closed wards in China. Its specific contribution is threefold: it foregrounds nurses as a professional group whose continuous bedside contact gives them distinctive influence over patient participation; it examines closed wards as a high-risk setting in which autonomy, coercion, and safety are negotiated daily; and it interprets nurses’ views not as simple knowledge deficits but as situated reasoning shaped by clinical risk, legal responsibility, family authority, and institutional support. The study therefore aims to clarify how psychiatric nurses understand SDM and how these understandings are translated into conditional support, cautious neutrality, or resistance in daily practice.

## 2 Methods

### 2.1 Study design

This study adopted a phenomenological qualitative design to explore psychiatric nurses’ lived understandings of patient participation in SDM. Phenomenology was chosen because the research question concerned the meanings, assumptions, emotional responses, and role interpretations through which nurses make sense of SDM in everyday closed-ward practice. A purely descriptive qualitative approach would have summarized opinions without sufficiently addressing lived experience, whereas grounded theory would have prioritized generation of a process theory beyond the study aim. The study followed the methodological logic of in-depth interviewing and interpretive thematic extraction, with Colaizzi’s seven-step analytical framework guiding the interpretation process (27). Reporting was checked against the Consolidated Criteria for Reporting Qualitative Research (COREQ) to strengthen transparency (28).

### 2.2 Setting and participants

The study was conducted between April and June 2024 in a tertiary Grade A psychiatric hospital in Jiangsu Province, China. Since 2023, the hospital had been exploring a decision-support system for patients with mental disorders and had introduced preliminary measures, including structured communication training, differentiated communication plans according to illness stage, and ward-level displays emphasizing respect for patients’ treatment decision rights. Purposive sampling with maximum variation was used to recruit nurses from closed acute, chronic, and rehabilitation wards. Inclusion criteria were possession of a valid nursing practice certificate, at least 5 years of psychiatric clinical nursing experience, current work in a closed psychiatric ward, adequate communication ability, and willingness to participate with signed informed consent. Adequate communication ability was operationally assessed during recruitment as the ability to understand the study information, converse fluently in Mandarin about routine clinical situations, provide coherent responses to the pre-interview screening questions, and complete a face-to-face interview without documented hearing, speech, or cognitive barriers that would prevent expression of professional experience. Exclusion criteria were internship or advanced training status and withdrawal during the interview process. The hospital had 382 psychiatric nurses in total. Eligible nurses were identified from the hospital information system and selected to maximize variation in sex, age, education, professional title, years of work, ward type, post, and shift pattern. Recruitment was conducted through written and oral invitation, participation was voluntary, and refusal or withdrawal had no effect on employment evaluation. Recruitment records contained no withdrawals after consent; non-participation among nurses in the wider eligible pool was not used as an analytic variable because the sampling strategy was purposive rather than enumerative. Recruitment stopped when informational saturation was reached, meaning that the final interviews repeatedly reproduced existing meanings, no additional codes altered the coding framework, and team discussion confirmed that further interviews were unlikely to generate a new theme. Sixteen nurses were ultimately included. Ethics approval was obtained from the hospital ethics committee (No. 2024-02). Written informed consent was obtained from all participants before enrollment, including consent for anonymized quotation.

### 2.3 Data collection

Data were collected through face-to-face, semi-structured, in-depth interviews. Before each interview, the investigator explained the study purpose, procedures, confidentiality arrangements, and voluntary nature of participation, after which written informed consent was obtained. The interview guide was drafted through literature review and team discussion, piloted with two nurses who were not included in the final analysis, and then refined for clarity and neutrality. The final guide explored participants’ understanding of SDM based on daily work experience, descriptions of SDM-related clinical experiences and the conclusions drawn from them, perceived facilitators and barriers if SDM were promoted in the hospital, nurses’ perceived roles in SDM scenarios, approaches to capacity and risk assessment, the role of family members, and participants’ professional attitude toward the participation of patients with mental disorders in SDM. The full interview guide is provided as **Supplementary Material 1**. At the end of each interview, participants were invited to add further reflections. Interviews were conducted by a senior head nurse with 20 years of psychiatric nursing experience who had completed systematic training in qualitative research. Interviews were conducted one participant per day in a quiet ward meeting room with comfortable light and temperature and minimal interruption. Interview duration ranged from 20 to 50 minutes. All interviews were audio-recorded, and non-verbal expressions such as gaze, facial expression, tone, and speech rate were documented in field notes. To protect privacy, participants were coded as N1 to N16.

### 2.4 Data analysis

Interview recordings were transcribed into text within 24 hours after each session and checked against audio recordings for accuracy. Data were analyzed using Colaizzi’s seven-step method: repeated reading of transcripts to obtain an overall sense; extraction of significant statements; formulation of meanings; clustering of meanings into themes; development of exhaustive descriptions; identification of the fundamental structure; and returning to participants or the research context for verification and refinement (27). NVivo 12.0 software was used to assist data management and coding. Coding was primarily inductive, while sensitizing concepts such as SDM, informed consent, autonomy, decision-making capacity, risk, and professional role were used during later interpretive comparison rather than imposed as a fixed coding template. Two researchers independently read the transcripts and conducted initial open coding. A preliminary coding framework was then discussed by the full research team, refined through memo writing and comparison of discrepant interpretations, and applied to the complete dataset. Disagreements were resolved through discussion until consensus was achieved, with original quotations, field notes, and contextual memos used to support decisions. Seventeen interview sessions were completed because one participant was interviewed twice at a one-week interval to clarify ambiguous statements about patient capacity, risk responsibility, and nurses’ role boundaries; the second interview was coded together with the first interview under the same participant code and used to refine existing categories rather than to create an additional case. The cumulative interview time was 447 minutes. Mechanical repetition and non-substantive responses were not coded; the analyzable transcripts comprised 42, 281 Chinese characters. A total of 262 significant statements were extracted, condensed into 62 codes, and synthesized into five themes that captured the major patterns in nurses’ understandings and attitudes.

### 2.5 Rigor and trustworthiness

Several procedures were used to enhance credibility, dependability, and confirmability. All members of the research team had received training in qualitative research and in the concept of shared decision-making. A pilot interview was conducted before formal data collection to ensure that the interview questions were understandable and relevant. Participants were recruited from closed wards so that the working environment would remain broadly comparable while still allowing variation in personal and professional background. An audit trail was maintained, including the interview guide, recruitment records, audio files, transcripts, field notes, coding framework, analytic memos, and minutes of team discussions. Formal member checking of all final themes was not undertaken; instead, credibility was supported through clarification questions during interviews, the follow-up interview with one participant, repeated comparison of codes with source quotations, and peer discussion within the research team.

Reflexivity was treated as a central issue because the interviewer was a senior head nurse and therefore occupied a professional position that could influence participants’ willingness to criticize institutional routines, coercive practices, risk management, or managerial expectations. The interviewer did not directly evaluate the participants’ annual performance during the research period, but the possibility of perceived authority could not be eliminated. To reduce conformity and social desirability bias, recruitment information emphasized that participation was voluntary, interview content would be anonymized, no individual comments would be reported to ward managers, and there were no correct or preferred answers. During interviews, the interviewer used neutral prompts, avoided evaluative feedback, and invited examples that contradicted the hospital’s current SDM initiatives. During analysis, reflexive memos were used to identify where the research team’s pro-SDM assumptions, nursing background, or familiarity with closed-ward routines might have led to overinterpretation. Interpretations that framed nurses’ concerns as simple resistance were reconsidered against field notes and quotations so that organizational culture, hierarchy, legal responsibility, and authority dynamics were incorporated into the final analysis.

## 3 Results

A total of 16 psychiatric nurses were included. Their characteristics are presented in **Table 1**. The sample covered both sexes, diploma to postgraduate education, junior to senior professional titles, 5 years to more than 20 years of psychiatric experience, staff nurses and head nurses, rotating and day shifts, and acute, chronic, and rehabilitation wards. This variation helped the study capture how SDM was understood across different positions within closed-ward work, although the sample was not designed for statistical subgroup comparison.

**Table 1:** Characteristics of the participants (N = 16).

ID	Sex	Age	Education	Title	Years	Ward	Post	Shift
N1	Female	24	Diploma	Junior	5–10	Acute ward	Staff nurse	Rotating
N2	Female	35	Bachelor	Intermediate	11–20	Chronic ward	Staff nurse	Rotating
N3	Female	49	Postgraduate	Senior	>20	Chronic ward	Head nurse	Day shift
N4	Female	27	Bachelor	Junior	5–10	Acute ward	Staff nurse	Rotating
N5	Male	38	Bachelor	Intermediate	11–20	Acute ward	Staff nurse	Rotating
N6	Female	34	Bachelor	Intermediate	11–20	Chronic ward	Staff nurse	Rotating
N7	Female	39	Bachelor	Intermediate	11–20	Rehabilitation ward	Staff nurse	Day shift
N8	Female	41	Bachelor	Senior	>20	Chronic ward	Staff nurse	Day shift
N9	Female	29	Bachelor	Intermediate	5–10	Acute ward	Staff nurse	Rotating
N10	Male	36	Bachelor	Intermediate	11–20	Rehabilitation ward	Staff nurse	Day shift
N11	Male	35	Bachelor	Intermediate	11–20	Chronic ward	Staff nurse	Rotating
N12	Female	31	Diploma	Intermediate	11–20	Chronic ward	Staff nurse	Rotating
N13	Male	26	Diploma	Junior	5–10	Acute ward	Staff nurse	Day shift
N14	Male	42	Bachelor	Intermediate	>20	Acute ward	Staff nurse	Day shift
N15	Female	30	Postgraduate	Intermediate	5–10	Rehabilitation ward	Head nurse	Day shift
N16	Female	42	Bachelor	Senior	>20	Acute ward	Head nurse	Day shift

**Figure 1:**
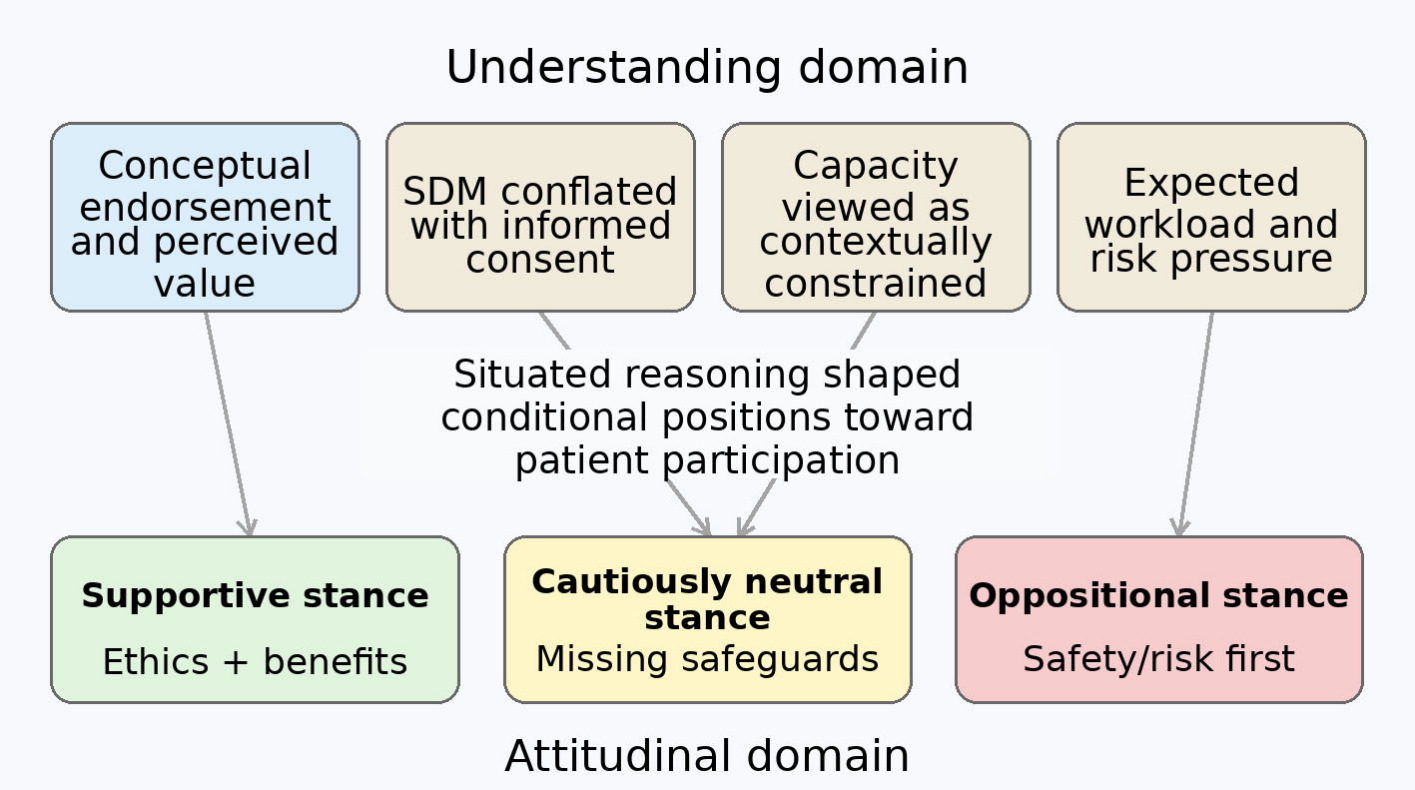
Thematic architecture of the understanding and attitudinal domains derived from the interview data.

Across the 17 interview sessions, the analysis yielded 262 significant statements and 62 preliminary codes, which were synthesized into five major themes. As shown in **Figure 1**, the findings were organized into two broad domains: understandings of patient participation in SDM and attitudinal positions toward that participation. Within the understanding domain, nurses recognized the ethical and clinical value of SDM but interpreted it through several forms of situated reasoning related to informed consent, patient capacity, workload, risk, and professional role. Within the attitudinal domain, nurses’ positions were fluid and conditional rather than fixed personality traits; movement between support, cautious neutrality, and opposition depended on patient acuity, decision type, ward culture, family involvement, institutional safeguards, and perceived responsibility.

The themes are summarized in **Table 2**. The first theme, conceptual endorsement and perceived clinical value, reflected broad agreement that SDM was compatible with patient-centred care. Participants described SDM as a way to respect disease experience, personal wishes, and individual differences. For example, N2 stated that SDM ‘respects patients’ disease experience, subjective wishes, and individual differences, ‘ while N5 explained that when patients are included, they become ‘more willing to talk about what they really feel.’ These accounts show that SDM was not viewed only as a rights discourse but also as a clinically useful strategy for improving communication, trust, and personalization of nursing plans.

**Table 2:** Themes and subthemes on psychiatric nurses’ understandings and attitudes toward SDM.

Domain	Theme	Analytic description
Understandings	Conceptual endorsement and perceived clinical value	Nurses viewed SDM as consistent with patient-centred care and believed it could improve communication, personalization, treatment cooperation, and therapeutic trust.
Understandings	SDM conflated with informed consent	Some participants described SDM as detailed explanation followed by patient agreement, preserving consent procedures but leaving limited space for joint option generation and deliberation.
Understandings	Decision-making capacity viewed as contextually constrained	Participants often linked patients’ ability to participate to illness phase, symptom acuity, chronic functional decline, safety risk, and the complexity of the decision.
Understandings	Expected workload and risk pressure	Many nurses anticipated that SDM would increase explanation time, documentation burden, conflict, and medico-legal exposure unless institutional support was strengthened.
Understandings	Nurses positioned as intermediaries rather than co-decision-makers	Nurses frequently described themselves as informants, executors, protectors, crisis interveners, or supporters rather than as professionals who actively sustain shared deliberation.
Attitude	Supportive position	Support was grounded in professional ethics, patients’ rights, and observed benefits such as improved adherence, stronger trust, and patient experiences of respect.
Attitude	Cautiously neutral position	Neutrality reflected guarded pragmatism in the absence of institutional safeguards, clear responsibility boundaries, and formal capacity or risk assessment pathways.
Attitude	Risk-focused oppositional position	Opposition centred on safety, urgency of treatment, involuntary or high-risk situations, and the belief that clinician-led decisions may be protective in specific contexts.

The remaining understanding themes reflected situated interpretations rather than simple errors. Some nurses conflated SDM with detailed explanation followed by consent. N7 described SDM as a situation in which the nurse explains a procedure, answers questions, and the patient ‘nods or signs after understanding it.’ This interpretation preserves informed consent but does not necessarily create joint deliberation about alternatives. Another theme concerned responsibility distribution: N11 understood SDM as letting patients take part so that ‘their own responsibility is also clear if problems occur later.’ This differs from role displacement because it concerns the purpose attributed to patient participation, whereas role positioning concerns how nurses locate themselves in the professional decision process. Capacity-related reasoning was also prominent. N8 argued that many chronic psychiatric patients had ‘deteriorated so much’ that they lacked the conditions to participate in decisions. These views were often linked to acute symptoms, chronic functional decline, or safety concerns, indicating that nurses treated capacity as constrained by context even when they did not explicitly distinguish task-specific capacity from global incapacity. Finally, many nurses anticipated that SDM would increase explanation time, documentation burden, conflict, and risk exposure, while others described nurses as information providers, executors, protectors, crisis interveners, or psychotherapy participants rather than core co-decision-makers. N6 summarized this role boundary by stating that ‘doctors must be dominant in SDM; nurses are supporting actors.’ Representative quotations illustrating these themes are shown in **Table 3**.

**Table 3:** Representative quotations from the interviews.

Theme	Participant	Illustrative quotation
Conceptual endorsement	N2	“SDM respects patients’ disease experience, subjective wishes, and individual differences, which is highly consistent with the nursing idea of patient-centred care.”
Clinical value	N5	“When patients are included in the decision process, they are more willing to talk about what they really feel, which helps us make more individualized plans.”
SDM conflated with informed consent	N7	“If I explain a procedure carefully, answer questions, and the patient nods or signs after understanding it, I think that should be SDM.”
Responsibility distribution	N11	“I understand SDM as letting patients take part so that their own responsibility is also clear if problems occur later.”
Capacity viewed as constrained	N8	“Many chronic psychiatric patients have withdrawn from society for years. Their thinking and cognition have deteriorated so much that I do not think they have the conditions to participate in decisions.”
Nurses positioned as intermediaries	N6	“Doctors must be dominant in SDM; nurses are supporting actors.”
Supportive position	N15	“After we invited a patient with bipolar disorder to participate in treatment and nursing planning, adherence improved a lot. She later said the process made her feel respected and safe.”
Cautiously neutral position	N7	“SDM may help, but there is still no mature support system. If there is no protection for staff, promoting it rashly brings professional risk.”
Risk-focused oppositional position	N14	“The professional team should make decisions and patients should cooperate with treatment. Giving patients too much decision responsibility may not help recovery.”

**Figure 2:**
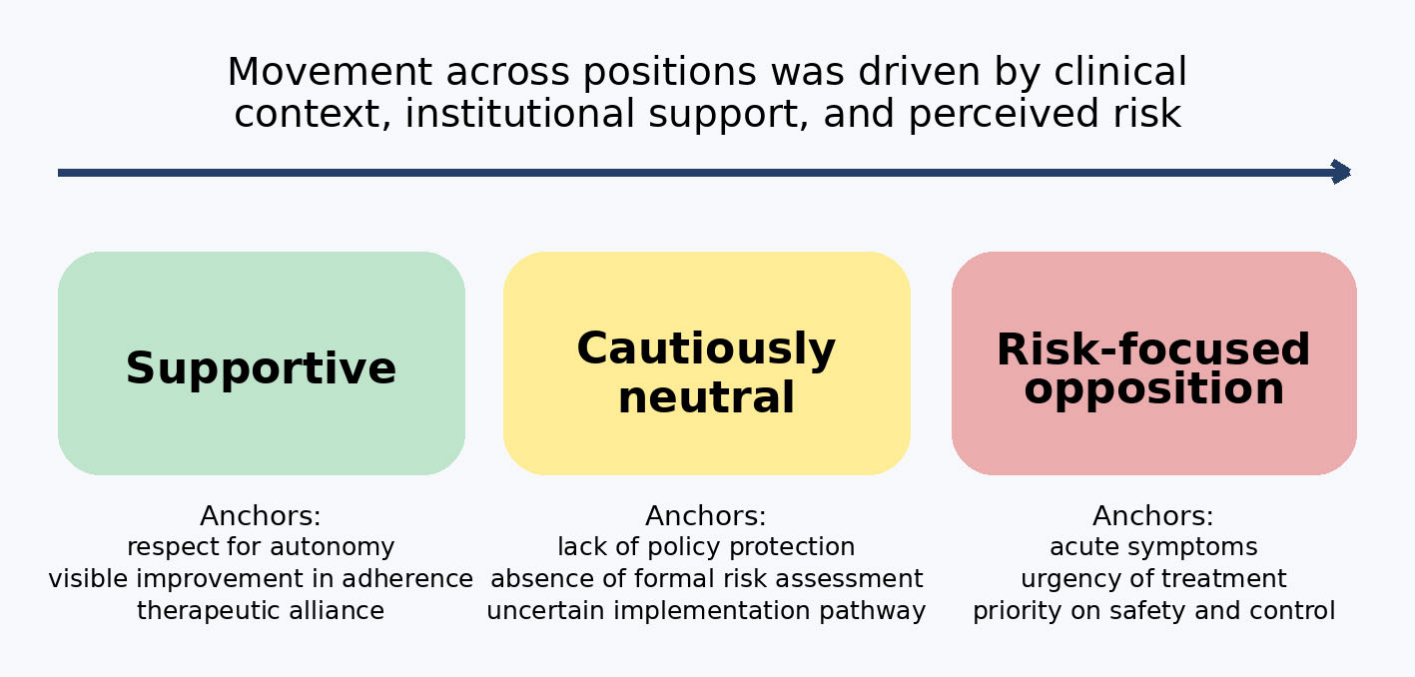
Conditional continuum of attitudinal positions toward implementing patient participation in SDM.

Attitudes toward implementation were heterogeneous and conditional. As depicted in **Figure 2**, supportive, cautiously neutral, and oppositional positions should be read as points on a continuum rather than as mutually exclusive categories. Supportive attitudes were grounded in professional ethics and concrete experiences in which participation improved cooperation, adherence, and feelings of being respected. N15 described a case in which a patient with bipolar disorder was invited to participate in treatment and nursing planning and subsequently reported feeling ‘respected and safe.’ Several nurses, including ward leaders, argued that supporting participation in decisions was not optional kindness but part of professional duty because patients had a legitimate right to be involved in matters affecting their treatment and recovery.

Cautiously neutral attitudes emerged among participants who did not reject SDM but questioned whether current institutional conditions were adequate for safe implementation. N7 noted that SDM ‘may help’ but argued that without a mature support system and staff protection, promoting it rashly would create professional risk. These nurses repeatedly referred to the absence of policy protection, unclear responsibility boundaries, and the lack of a formal risk-assessment pathway that could guide when and how patient participation should be supported in psychiatric wards. Their neutrality therefore reflected guarded pragmatism rather than ideological resistance. By contrast, risk-focused opposition was expressed by participants who considered safety and rapid treatment to be overriding principles in particular circumstances. N14 stated that ‘the professional team should make decisions and patients should cooperate with treatment, ‘reflecting a belief that excessive decision responsibility may not help recovery when symptoms, risk, or urgency are prominent. For these nurses, clinician-led decisions were interpreted as a form of protection rather than merely as paternalism.

Clinical context and participant characteristics shaped these positions in interpretive rather than statistically comparable ways. Nurses from acute wards more often emphasized symptom acuity, rapid intervention, aggression or self-harm risk, and the difficulty of prolonged deliberation. Nurses from chronic wards more often referred to long illness duration, social withdrawal, family responsibility, and doubts about sustained decisional engagement. Nurses from rehabilitation wards more readily discussed daily-life goals, medication routines, and recovery planning as areas where conditional participation was feasible. Differences by professional title or seniority were less stable: senior nurses and head nurses sometimes voiced stronger rights-based support because of management experience with ward initiatives, but they also expressed sharper concern about liability and risk responsibility. These patterns suggest that attitudes toward SDM were context-sensitive and ambivalent rather than fixed by a single demographic attribute.

## 4 Discussion

This study shows that psychiatric nurses in closed wards generally accepted the moral legitimacy of patient participation in SDM, yet their everyday understandings were conditional and shaped by the institutional realities of psychiatric care. This tension between conceptual approval and practical hesitation has also been reported in recent mental health literature, where staff members describe SDM as desirable in principle but difficult to operationalize under real-world constraints (3–6, 10–12). The present findings extend that literature by showing how closed-ward nurses translate abstract support for participation into situated judgments about capacity, risk, family involvement, role boundaries, and legal responsibility. Thus, the issue is not only whether nurses know the formal definition of SDM, but how they decide whether participation is clinically meaningful and safe in a specific ward situation.

A first implication concerns conceptual precision. When nurses equated SDM with careful explanation and agreement, they preserved an important component of ethical care but reduced SDM to informed consent. Informed consent is necessary before many interventions, yet it does not by itself ensure that patients help shape options, express preferences, or participate in deliberation. Similar tensions have been observed in qualitative studies showing that staff may use partnership language while retaining a predominantly clinician-led decision architecture (4, 10, 15, 16). However, these accounts should not be read simply as professional errors. In closed psychiatric wards, careful explanation and signed agreement are highly visible forms of procedural protection, and nurses may understandably rely on them when responsibility boundaries are unclear. Training should therefore differentiate SDM from informed consent, compliance counselling, and defensive documentation while acknowledging why these practices become attractive in high-risk institutional contexts.

A second issue concerns decision-making capacity. Participants’ capacity-related concerns could reflect paternalism, limited SDM training, or diagnostic generalization, but they may also reflect legitimate clinical experience with fluctuating symptoms, impaired insight, acute risk, and rapidly changing decisional ability. Current scholarship emphasizes that decisional capacity is task-specific and situation-dependent rather than equivalent to diagnosis alone (13). The present data suggest that in daily ward practice, visible symptoms, chronic functional decline, or urgent safety concerns may still operate as shorthand for broad incapacity. Such reasoning can become exclusionary when it prevents patients from being invited into decisions that could be supported, staged, or revisited. The implication is not that all patients should decide everything independently, but that capacity assessment and SDM should be matched to decision type, illness phase, and available support. Structured assessment and tailored decision support are therefore more appropriate than blanket exclusion, especially given emerging work on SDM measures for people with limited decisional capacity (7, 9, 13, 17, 29).

The study also highlights how organizational pressure shapes attitude. Nurses’ expectations that SDM would increase workload, conflict, and medico-legal exposure were clinically meaningful because closed psychiatric wards operate under staffing constraints, symptom volatility, and safety demands. Nevertheless, literature on SDM implementation suggests that professionals may overestimate front-end communication costs while underestimating downstream benefits such as improved adherence, reduced conflict, and better alignment between care plans and patient expectations (6, 8, 14, 19, 23). In this study, nurses who had observed beneficial cases were more likely to support SDM, suggesting that visible positive experience may be a stronger implementation lever than abstract ethical instruction alone. Hospitals should therefore create mechanisms through which successful and unsuccessful SDM cases are discussed, documented, and translated into locally credible examples.

A further contribution of the study lies in its clarification of role ambiguity in psychiatric nursing. Participants could list many auxiliary functions in the decision process but often positioned themselves as informants, executors, or protectors rather than as active co-decision-makers. This pattern is important because nurses are frequently the professionals who sustain deliberation over time, interpret patient concerns in everyday language, and recognize when a formally accepted treatment plan is not subjectively acceptable to the patient. Recent literature has argued that nursing professionals can be key actors in SDM precisely because of their relational continuity with service users (18–21). The current findings suggest that this potential remains under-realized when institutional culture frames doctors as decision leaders, families as final authorities, and nurses as intermediaries. Strengthening nurses’ role confidence and clarifying interprofessional responsibility may therefore be as important as improving patients’ participation skills.

The attitudinal continuum identified in this study should be interpreted critically rather than normatively. Supportive, cautiously neutral, and oppositional positions were not simply indicators of good or poor professional attitudes. Neutral and oppositional positions often expressed a serious commitment to patient safety, legal responsibility, and feasible practice. Nurses working in closed wards are exposed to aggression risk, medication refusal, self-harm risk, involuntary treatment, and episodes where delayed intervention can have severe consequences. In such environments, professional caution can become a default moral orientation. The problem arises when safety is treated as automatically incompatible with participation, because patients may then be excluded from decisions that could in fact be discussed in partial, supported, or temporally staged ways. A realistic SDM model for psychiatric wards must therefore integrate coercion, risk management, capacity fluctuation, and clinical responsibility rather than treating them as external barriers to an otherwise stable ideal.

Several practice implications follow. First, implementation should be differentiated by illness phase and ward context. In acute wards, SDM may begin with narrow but meaningful elements such as eliciting immediate concerns, identifying unacceptable options, explaining the reasons for urgent interventions, and revisiting decisions once symptoms stabilize. In chronic and rehabilitation wards, broader negotiation around medication routines, rehabilitation goals, family communication, discharge preparation, and daily care plans may be more feasible. Second, institutions need formal support structures, including protocols that specify capacity and risk assessment pathways, documentation standards for shared deliberation, escalation procedures when disagreement intersects with risk, and clear boundaries of professional accountability. Third, structured SDM training should be scenario-based and include capacity-sensitive communication, de-escalation language, family negotiation, recognition of coercive drift, and approaches for documenting patient preferences even when the final decision remains clinician-led for safety reasons. Fourth, interprofessional case discussions should include nurses, psychiatrists, psychologists, social workers, patients, and family members when appropriate, so that responsibility is distributed and patient participation does not become a burden carried by a single professional. Fifth, decision aids adapted to psychiatric nursing could provide a practical structure for repeated conversations about options, benefits, burdens, relapse warning signs, and recovery goals (23, 24, 29, 30).

This study has limitations. It was conducted in a single tertiary psychiatric hospital in Jiangsu Province, and all participants worked in closed wards; therefore, the findings may not transfer directly to open wards, community settings, private hospitals, or psychiatric systems with different legal and cultural arrangements. The focus on closed wards strengthened the study’s attention to autonomy, coercion, capacity, and safety, but it also means that the findings may overrepresent risk-focused reasoning compared with settings serving patients with milder conditions. Purposive sampling may have introduced selection bias because nurses willing to discuss SDM could differ from those who were not approached or who did not participate in the wider eligible pool. The interviewer was a senior nurse manager, and participants’ responses may have been shaped by perceived hierarchy, organizational culture, conformity, or social desirability despite confidentiality safeguards. Formal member checking of the final themes was not systematically performed, which limits the extent to which participants could validate the interpretive structure. Because the study was conducted in Chinese and the quotations were translated into English, some linguistic and cultural nuance may have been lost. Finally, Chinese legal norms, family involvement, and institutional responsibility arrangements may limit direct transferability to countries where autonomy, guardianship, involuntary treatment, and family authority are structured differently.

## 5 Conclusion

Psychiatric nurses in this study generally endorsed respect for patients with mental disorders in clinical decision-making, but their support for SDM was conditional rather than uniform. Their understandings and attitudes were shaped by how SDM was distinguished from informed consent, how fluctuating decision-making capacity was interpreted, how workload and risk responsibility were anticipated, how nurses positioned their own professional role, and how closed-ward culture balanced patient participation with safety, coercion, family authority, and legal responsibility. These findings suggest that SDM in psychiatric nursing should be implemented not as a universal slogan but as a capacity-sensitive, risk-aware, and institutionally supported practice adapted to illness phase and ward context.

## Data Availability

The raw data supporting the conclusions of this article will be made available by the authors, without undue reservation.
