# Supplementary material for: Psychiatric nurses’ understandings and attitudes toward the participation of patients with mental disorders in shared decision-making: a phenomenological study
Source: Front Psychiatry. 2026 Jul 17;17:1864939. doi: 10.3389/fpsyt.2026.1864939 (PMC13423842; doi:10.3389/fpsyt.2026.1864939)
Supplement: Supplementary file 1 [file Supplementaryfile1.docx]

Supplementary Material 1. Semi-structured interview guide

This guide was used flexibly during face-to-face interviews. Follow-up prompts were adapted to participants’ answers while maintaining neutrality and avoiding evaluative feedback.

1. Please describe how you understand shared decision-making in psychiatric nursing practice.

2. In your daily work, what kinds of decisions do you think patients with mental disorders can participate in, and what kinds of decisions are more difficult for them to participate in?

3. Please recall a clinical situation in which a patient participated, attempted to participate, or was unable to participate in a treatment or nursing decision. What happened, and what did you think about it?

4. How do you usually judge whether a patient can understand information, express preferences, or participate in a specific decision?

5. How do illness phase, symptom severity, risk of harm, urgency of treatment, and ward safety influence your attitude toward patient participation?

6. What roles should nurses, psychiatrists, patients, and family members play in shared decision-making in closed psychiatric wards?

7. What institutional supports, procedures, training, or decision aids would be needed if SDM were further implemented in your ward?

8. Overall, what is your attitude toward promoting participation of patients with mental disorders in SDM, and what are the main reasons for this attitude?
